# Unravelling diagnostic clusters and immune landscapes of disulfidptosis patterns in gastric cancer through bioinformatic assay

**DOI:** 10.18632/aging.205365

**Published:** 2023-12-27

**Authors:** Peng Zhang, Zhuofeng Chen, Xiaocheng Lin, Siyao Yu, Xiang Yu, Zhuoqun Chen

**Affiliations:** 1 Guangzhou University of Chinese Medicine, Guangzhou 510405, China; 2 The First Affiliated Hospital of Shantou University Medical College, Shantou 515041, China; 3 Macalester College, Saint Paul, MN 55105, USA; 4 The First Affiliated Hospital of Guangzhou University of Chinese Medicine, Guangzhou 510405, China; 5 Guangdong Clinical Research Academy of Chinese Medicine, Guangzhou 510405, China

**Keywords:** gastric cancer, disulfidptosis modulators, risk prediction, subtype classification, immune cell infiltration

## Abstract

Disulfidptosis is a novel type of cell death mediated by SLC7A11-induced disulfide stress. Gastric cancer (GC) is a common malignant gastrointestinal tumor. Existing evidence shows that SLC7A11 can regulate cell death and improve the progression of GC, suggesting disulfidptosis may exist in the pathological process of GC. However, the underlying functions of disulfidptosis regulators in GC remain unknown. The dataset of GSE54129 was screened to comprehensively investigate the disulfidptosis-related diagnostic clusters and immune landscapes in GC. Totally 15 significant disulfidptosis regulators were identified via difference analysis between GC samples and controls. Then random forest model was utilized to assess their importance score (mean decrease Gini). Then a nomogram model was constructed, which could offer benefit to patients based on our subsequent decision curve analysis. All the included GC patients were divided into 2 disulfidptosis subgroups (clusterA and clusterB) according to the significant disulfidptosis regulators in virtue of consensus clustering analysis. The disulfidptosis score of each sample was calculated through PCA algorithms to quantify the disulfidptosis subtypes. Patients from clusterB exhibited lower disulfidptosis scores than those of patients in clusterA. In addition, we found that the cases in clusterB were closely associated with the immunity of activated CD4 T cell, etc., while clusterA was linked to immature dendritic cell, mast cell, natural killer T cell, natural killer cell, etc., which has a higher disulfidptosis score. Therefore, disulfidptosis regulators play an important role in the pathological process of GC, providing a promising marker and an immunotherapeutic strategy for future GC therapy.

## INTRODUCTION

Gastric cancer (GC) is a common malignant tumor of the gastrointestinal tract with about 1.1 million new cases each year and even 800 thousand people dying of gastric cancer each year, ranking 4th among the causes of death from malignant tumors [1, 2]. Clinical management of GC is not satisfactory nowadays, and existing medicinal products including perbrolizumab, ramucirumab and apatinib have relatively serious side effects such as heart dysfunction, blood toxicity and certain liver and kidney function damage [3]. GC poses great threat to patients’ health and life quality, and even reduces their life expectancy, raising the cost of health care and the financial burden on families and societies [4]. Hence, early identification of patients at high risk of GC is essential and vital. Increasing evidence from the extensive development of GC research suggests that GC is a complex disease with great heterogeneity and genetic variation [5]. Therefore, early precaution and identification of patients at high risk of developing GC from a genetic point of view will have a profound influence on the control of GC epidemiology.

Disulfidptosis is a disulfide stress-induced manner of cell death, which occurs under the condition of glucose starvation that the NADPH in cells with unbalanced expression of SLC7A11 was consumed, and the abnormal accumulation of cystine and other disulfide compounds induced disulfide stress and rapid cell death [6]. As a distinct cell death mechanism, disulfidptosis needs numerous disulfide-regulatory proteins including SLC7A11, SLC3A2, FLNA, FLNB, MYH9, TLN1, ACTB, PRDX1, RPN1, NUBPL, NCKAP1, LRPPRC, NDUFA11, NDUFS1, OXSM, GYS1, WAVE2 and RAC1 to cooperate together [6]. Importantly, existing reports have confirmed that diallyl disulfide plays an important part in inhibiting tumor growth activity in GC [7]. Moreover, SLC7A11 has been reported to reversely regulate ferroptosis of GC cells and thus promote the progression of GC, indicating the promise of sulfide-related proteins as potential targets in the diagnosis and treatment of GC [8]. Therefore, we speculate that disulfidptosis has an important role in the pathological process of GC via regulating disulfide stress-associated gene expression. However, the role of disulfidptosis regulators in GC remains unclear.

**Figure 1:**
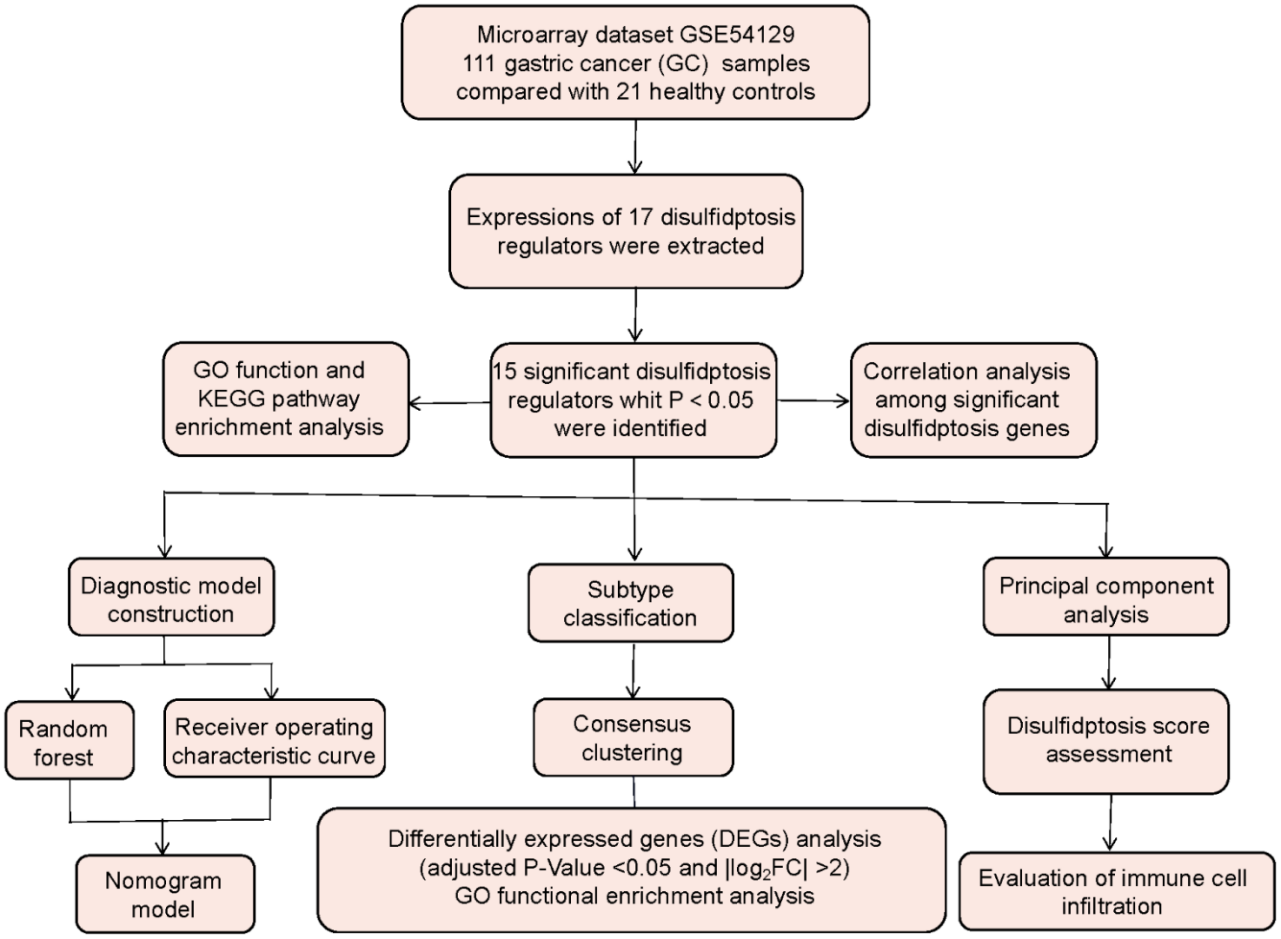
Flow chart of the study design.

On the basis of the GSE54129 dataset, we evaluated disulfidptosis regulators’ contributions to the discovery of GC subtypes and diagnostic biomarkers. We created a gene model for GC susceptibility based on 15 potential disulfidptosis regulators including FLNA, FLNB, MYH9, TLN1, ACTB, PRDX1, SLC7A11, SLC3A2, RPN1, NCKAP1, NDUFA11, OXSM, NDUFS1, GYS1, RAC1, and discovered that the model offered patients good clinical advantages. Notably, we discovered two distinct disulfidptosis patterns that were highly correlated with abundant immune cell infiltration, suggesting that disulfidptosis patterns may be used to diagnose GC and offer follow-up treatment options. Moreover, we examined the correlation between disulfidptosis patterns and IL6, and IL33 which have close association with natural killer cell immunity in GC. Figure 1 displayed the flowchart of study design and process.

## MATERIALS AND METHODS

### Retrieval of GC samples

Using the GEO database (http://www.ncbi.nlm.nih.gov/geo/), we obtained GC samples isolated from gastric cancer tissues of GC patients. “Gastric cancer”, “Gene expression”, and “Microarray” were the search phrases, and the datasets were chosen according to the eligibility principles: the dataset has at least 40 samples containing at least 20 cases respectively in the control and GC groups, which also provides both raw data and series matrix files available to download. Finally, the dataset GSE54129 was reviewed, and it totally satisfied our requirements. Using this dataset, we selected 21 noncancerous cases from the control group and 111 cases from the GC group for further study.

### Data collection

To convert microarray probes to symbols, we obtained the annotated R package (v4.1.2) from Bioconductor (http://bioconductor.org/). Following data preparation, we used quantile normalization to further standardize the data, which included 21 controls and 111 GC cases. Distinct disulfidptosis regulators were determined in the dataset by difference analysis of controls and GC patients with the R package of Limma. The *P*-Value<0.05 and |log_2_ fold change (FC)|>0 were used as screening thresholds to discover the significant disulfidptosis regulators [9]. Thereafter, we used the R package “clusterProfiler” and the Enrichr platform (https://maayanlab.cloud/Enrichr/) to conduct GO and KEGG enrichment analysis to study the probable mechanism of the disulfidptosis regulators implicated in GC.

### Model establishment

To analyze the occurrence of GC, we built a random forest (RF) model using the R package “RandomForest” to select putative disulfidptosis mediator with highest significance score (Mean Decrease Gini), which was then chosen for constructing “Receiver operating characteristic (ROC) curve” [10]. Thereafter, the R package of “rms” was utilized to construct a nomogram model to predict the prevalence of GC patients according to screened candidate disulfidptosis regulator. Calibration curves were utilized to determine how well the prediction values match reality. Decision curve analysis (DCA) was used to create a clinical impact curve and determine if decisions based on the model benefited patients [11].

### Subtype identification

Using consensus clustering relying on resampling, each member and its subcluster number are identified, validating that the clusters are rational [11]. Based on significant disulfidptosis regulators, the “ConsensusClusterPlus” R package was used to identify different disulfidptosis patterns [12].

### GO enrichment analysis of differentially expressed genes between different disulfidptosis clusters

Using the Limma package, we identified differentially expressed genes (DEGs) between different disulfidptosis clusters using a threshold of adjusted *P*-Value <0.05 and |log_2_FC| >2. GO analysis was then performed using “clusterProfiler” R package to elucidate how DEGs may participate in the process of GC [13].

### Disulfidptosis score assessment

The disulfidptosis score for each sample was calculated using principal component analysis (PCA), aiming to quantify the disulfidptosis patterns. This score was calculated using the following formula: disulfidptosis score = PC1_i_, where PC1 represents principal component 1, and i represents significant disulfidptosis gene expression [14].

### Immune cell infiltration assessment

The quantity of immune cell infiltration in GC group samples was assessed using single sample gene set enrichment analysis (ssGSEA). To begin with, ssGSEA was used to sequence the gene expression levels in the samples in order to generate a gene expression ranking. Thereafter, we looked through the input dataset for important regulators of disulfidptosis and aggregated their levels of expression. We obtained the amount of immune cells in each sample based on these analyses [15].

### Statistical analysis

Linear regression analyses were employed to evaluate the correlations among significant disulfidptosis genes. Kruskal-Wallis tests were used for group-wise comparisons in bioinformatics analysis. All parametric analyses went through two-tailed tests, with *P*<0.05 indicating statistical significance. The results were reported as mean ± standard deviation.

### Data availability statement

All the data from the present study can be obtained from the first author on reasonable request.

## RESULTS

### Screening of the 17 disulfidptosis regulators in GC

**Figure 2:**
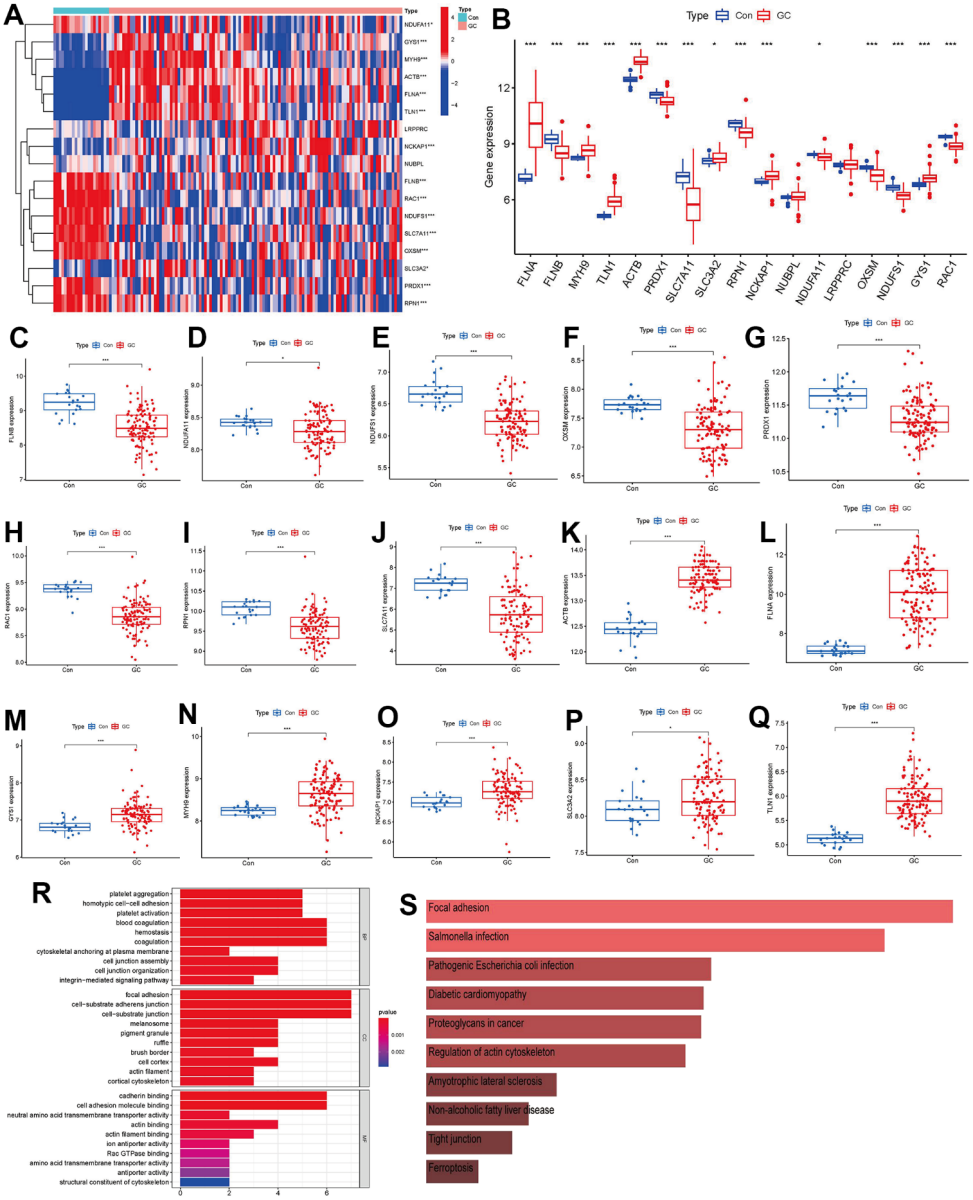
**Identification of the 17 disulfidptosis modulators in GC.** (**A**) Expression heat map of the 17 disulfidptosis modulators in controls and GC cases. (**B**) Differential expression boxplot of the 17 disulfidptosis modulators between controls and GC cases. (**C**–**Q**) Differential expression boxplot of 15 significant disulfidptosis modulators identified between controls and GC cases. (**R**–**S**) GO and KEGG enrichment analysis based on the 15 significant disulfidptosis modulators. *p < 0.05, and ***p < 0.001.

A total of 17 disulfidptosis regulators were determined through gene expressions between difference from the two groups of controls and GC cases. We ultimately discovered 15 distinct disulfidptosis regulators (FLNA, FLNB, MYH9, TLN1, ACTB, PRDX1, SLC7A11, SLC3A2, RPN1, NCKAP1, NDUFA11, OXSM, NDUFS1, GYS1, RAC1), which were shown in a heat map and boxplot (Figure 2A, 2B). We observed that the expressions of ACTB, FLNA, GYS1, MYH9, NCKAP1, SLC3A2 and TLN1 were increased in GC cases compared to controls, but the other significant regulators of disulfidoptosis exhibited opposite outcomes (Figure 2C–2Q). We observed that GO enrichment results including biological process (cytoskeletal anchoring at plasma membrane), cellular components (cortical cytoskeleton), molecular function (structural constituent of cytoskeleton) were the mainly enriched entries (Figure 2R). Notably, KEGG enrichment analysis showed that the regulations of actin cytoskeleton and ferroptosis were the mainly enriched pathways (Figure 2S). The detailed information of GO and KEGG enrichment analysis was shown in Supplementary Tables 1, 2.

### Correlation among disulfidptosis modulators in GC

**Figure 3:**
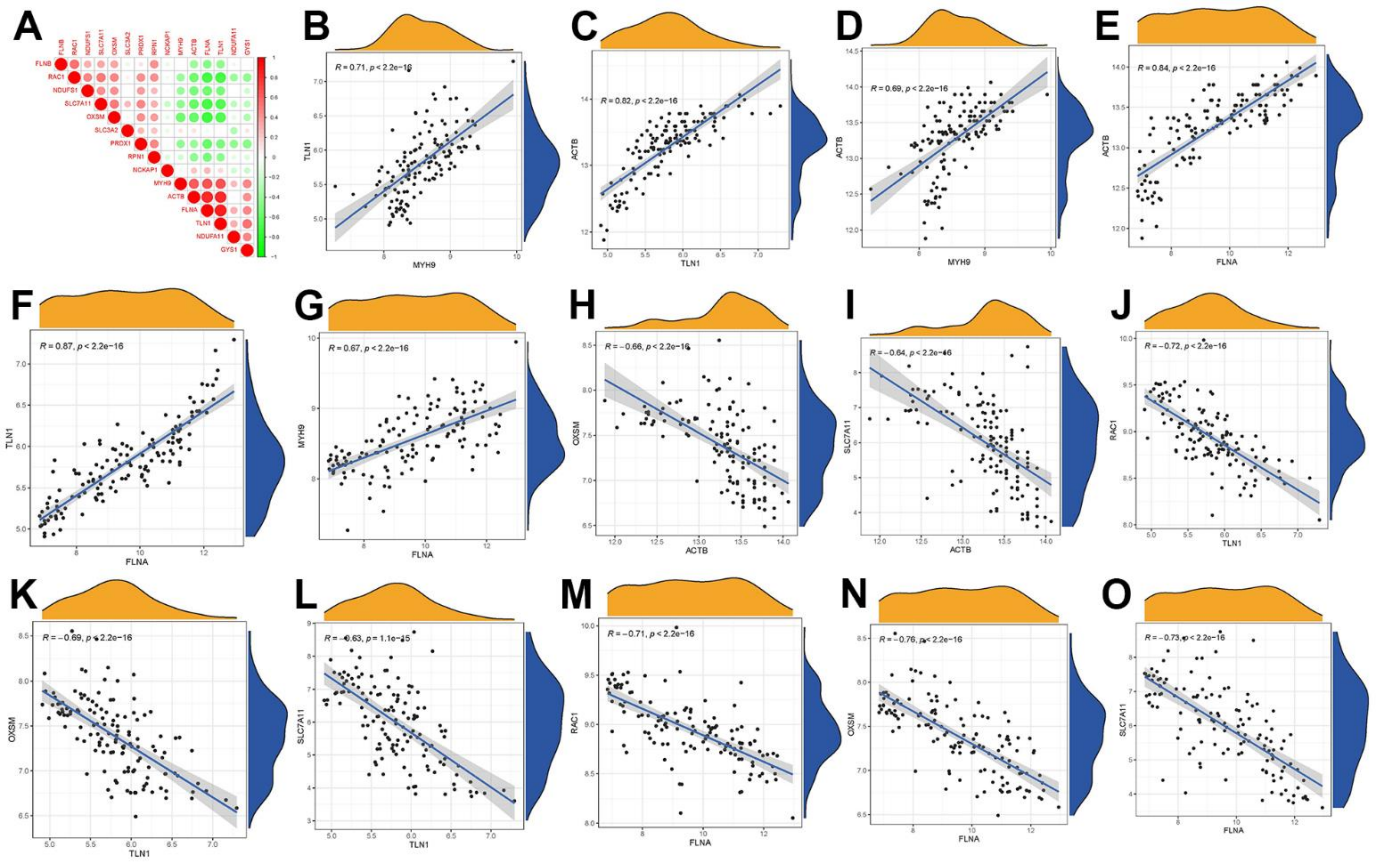
**Correlation among disulfidptosis modulators in GC.** (**A**) Correlation heat map of different correlations between different disulfidptosis modulators. There existed significantly positive correlations in the gene expression levels of TLN1-MYH9, ACTB-TLN1, ACTB-MYH9, ACTB-FLNA, TLN1-FLNA, MYH9-FLNA in GC cases (**B**–**G**), while the gene expression levels of OXSM-ACTB, SLC7A11-ACTB, RAC1-TLN1, OXSM-TLN1, SLC7A11-TLN1, RAC1-FLNA, OXSM-FLNA, SLC7A11-FLNA in GC cases exhibited significantly negative correlation (**H**–**O**).

In Figure 3A, we demonstrated different correlations between different disulfidptosis modulators in GC. Then, we screened the significant correlations with |R|>0.6 for visualization. There existed significantly positive correlations in the gene expression levels of ACTB-TLN1, TLN1-MYH9, ACTB-MYH9, ACTB-FLNA, MYH9-FLNA, TLN1-FLNA in GC cases (Figure 3B–3G), while the gene expression levels of OXSM-ACTB, SLC7A11-ACTB, RAC1-TLN1, OXSM-TLN1, SCL7A11-TLN1, RAC1-FLNA, OXSM-FLNA, SLC7A11-FLNA in GC cases exhibited significantly negative correlation (Figure 3H–3O).

### Construction of the RF model

**Figure 4:**
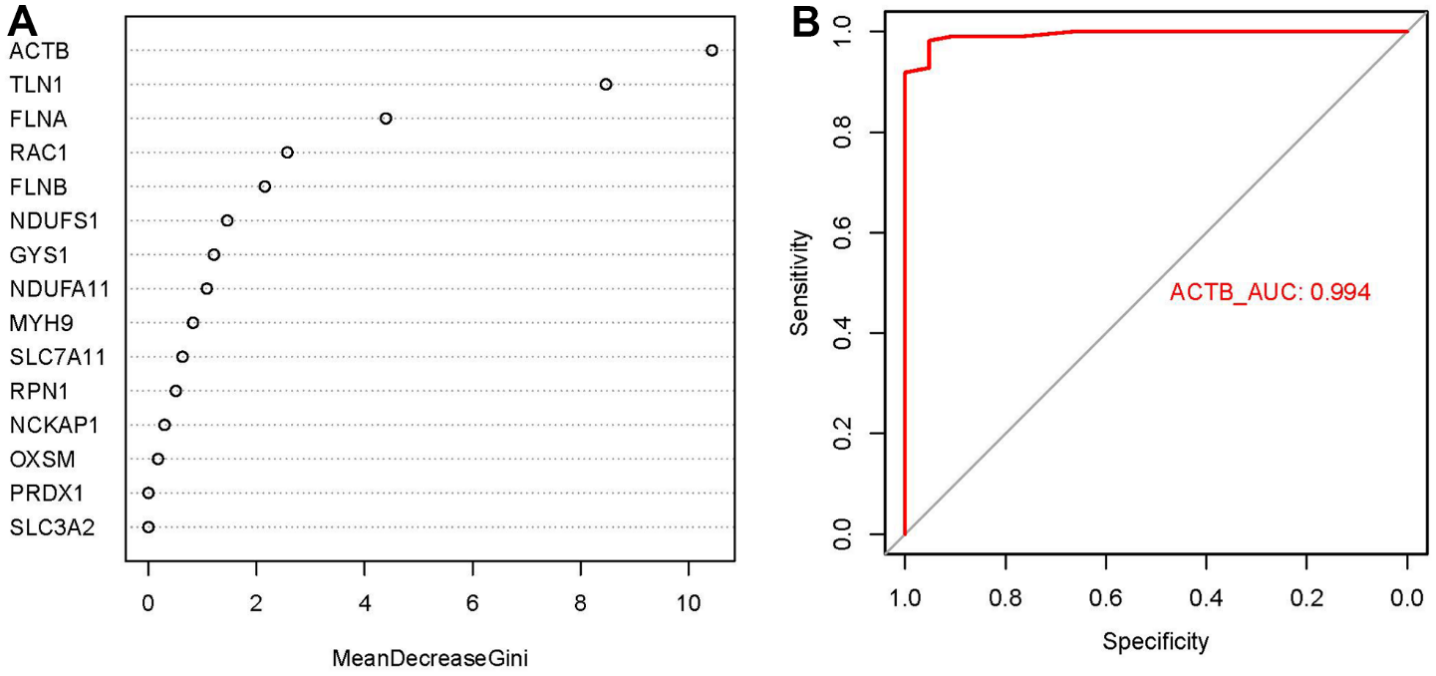
**Importance score and ROC curves assessed by RF model.** (**A**) The importance score of the 15 disulfidptosis modulators on the basis of the RF model. (**B**) ROC curves indicated ACTB with the AUC value of 0.994.

We presented these 15 significant disulfidptosis regulators after ranking them in order of importance score (mean decrease Gini) and selected ACTB with the highest importance score as the hub gene (Figure 4A). Thereafter, we used ROC curves to estimate the model, and the AUC value of ACTB was 0.994, indicating that the RF model has high accuracy to predict the occurrence of GC (Figure 4B).

### Nomogram model construction

**Figure 5:**
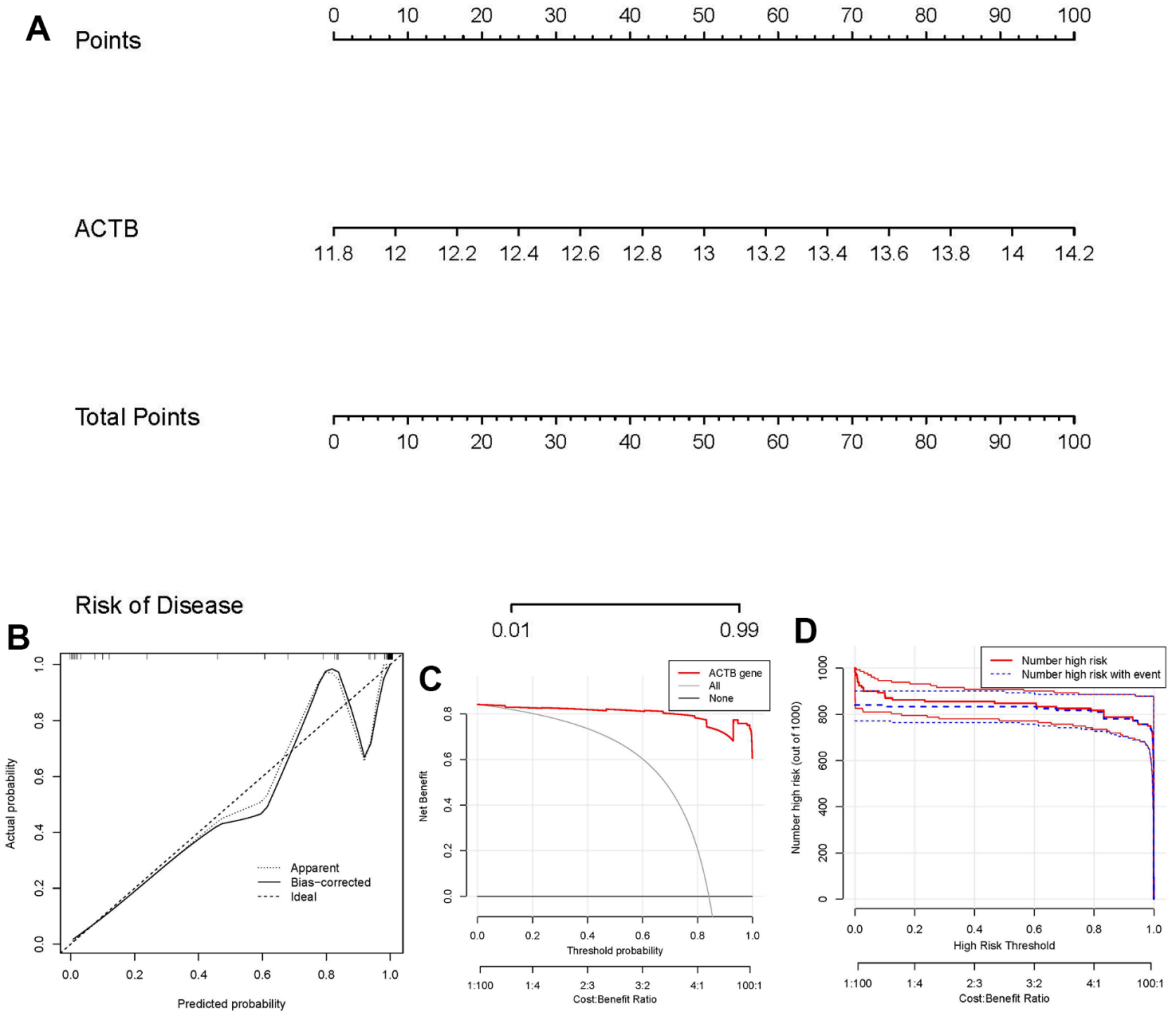
**Establishment of the nomogram model.** (**A**) The nomogram model was established on the basis of ACTB. (**B**) The calibration curve was utilized to evaluate the predictive accuracy of the nomogram model. (**C**) Decisions on the basis of this nomogram model may be beneficial to GC patients. (**D**) The clinical impact curve was used to assess clinical impact of the nomogram model.

For the purpose of predicting the prevalence of GC patients, we created a nomogram model of the hub gene ACTB using the “rms” package in R (Figure 5A). We found that the nomogram model demonstrated great prediction accuracy based on calibration curves (Figure 5B). From 0 to 1, the red line in the DCA curve continued to be higher than the gray and black lines, indicating that GC patients may benefit from judgments made using the nomogram model (Figure 5C). Additionally, we observed that the predictive capacity of the nomogram model was remarkable on the basis of the clinical impact curve (Figure 5D).

### Recognition of two different disulfidptosis clusters

**Figure 6:**
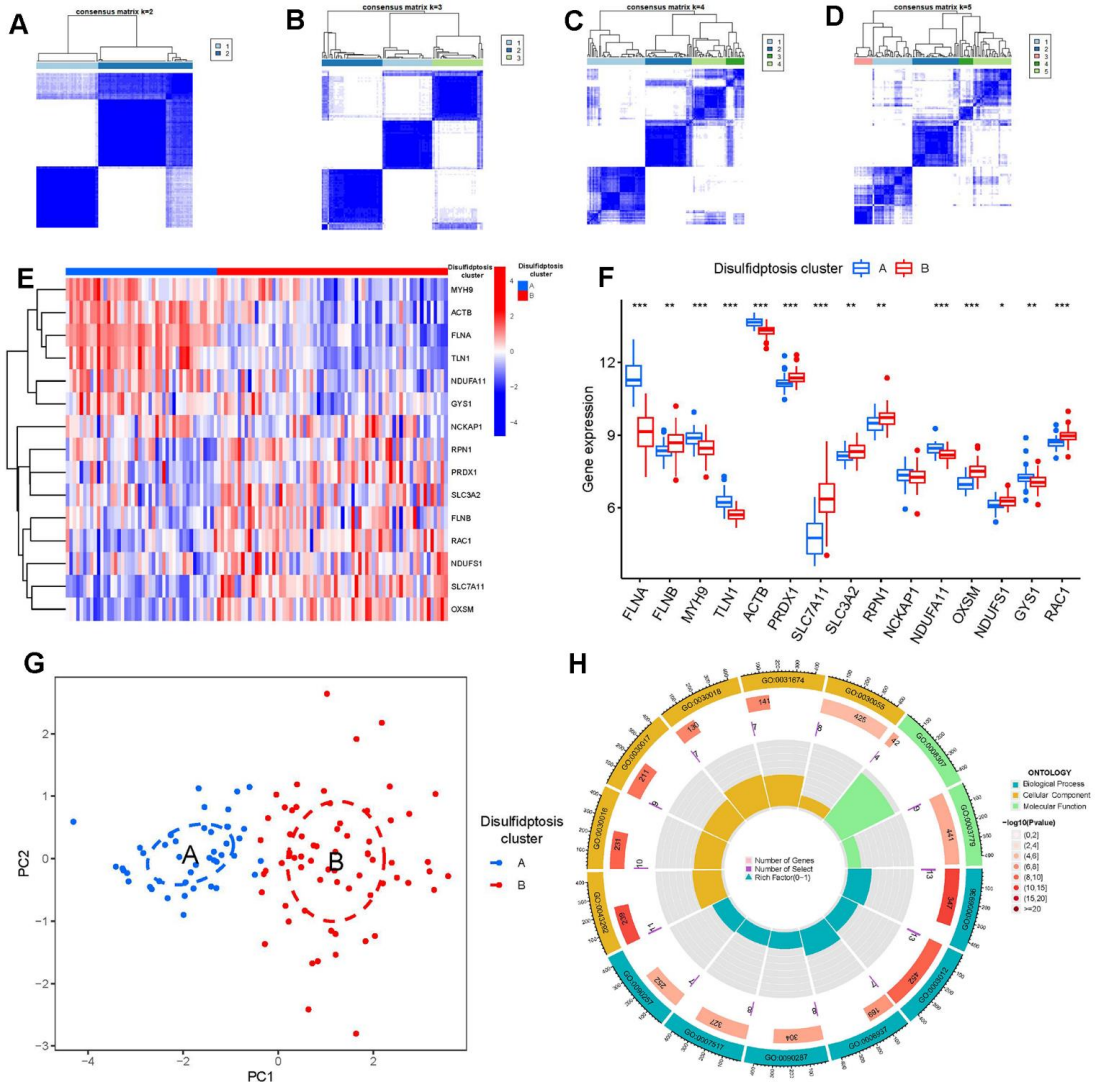
**Consensus clustering of the 15 significant disulfidptosis modulators in GC.** (**A**–**D**) Consensus matrices of the 15 significant disulfidptosis modulators for k = 2–5. (**E**) Expression heat map of the 15 significant disulfidptosis modulators in clusterA and clusterB. (**F**) Differential expression boxplots of the 15 significant disulfidptosis modulators in clusterA and clusterB. (**G**) Principal component analysis for the expression profiles of the 15 significant disulfidptosismodulators that shows a remarkable difference in transcriptomes between the two disulfidptosis patterns. (**H**) GO enrichment analysis that explores the potential mechanism underlying the effect of the 79 disulfidptosis-related DEGs on the occurrence and development of GC. *p < 0.05, **p < 0.01, and ***p < 0.001.

Using the R package “ConsensusClusterPlus,” we discovered two disulfidptosis clusters (clusterA and clusterB) based on the 15 important disulfidptosis regulators. (Figure 6A–6D). Cluster A had 44 samples, whereas cluster B contained 67 samples. Thereafter, the differential expression levels of the 15 crucial disulfidptosis regulators between the two clusters were then clearly shown by the heat map and boxplot. We found that FLNA, MYH9, TLN1, ACTB, NDUFA11, GYS1 had higher expression levels in clusterA than in clusterB, while FLNB, PRDX1, SLC7A11, SLC3A2, RPN1, OXSM, NDUFS1, RAC1 had higher expression levels in clusterB than in clusterA. NCKAP1, however, did not show any discernible differences in expression levels between the two clusters (Figure 6E, 6F). The 15 important disulfidptosis regulators were found to be able to discriminate between the two disulfidptosis clusters according to the PCA findings (Figure 6G). Between the two disulfidptosis patterns, we identified 79 DEGs linked with disulfidptosis. To further understand the function of these DEGs in GC, we performed GO enrichment analysis (Figure 6H). The detailed information of GO enrichment analysis was shown in Supplementary Table 3. We observed that GO:0090287 (regulation of cellular response to growth factor stimulus), GO:0030055 (cell-substrate junction) and GO:0003779 (actin binding) were the mainly enriched entries.

**Figure 7:**
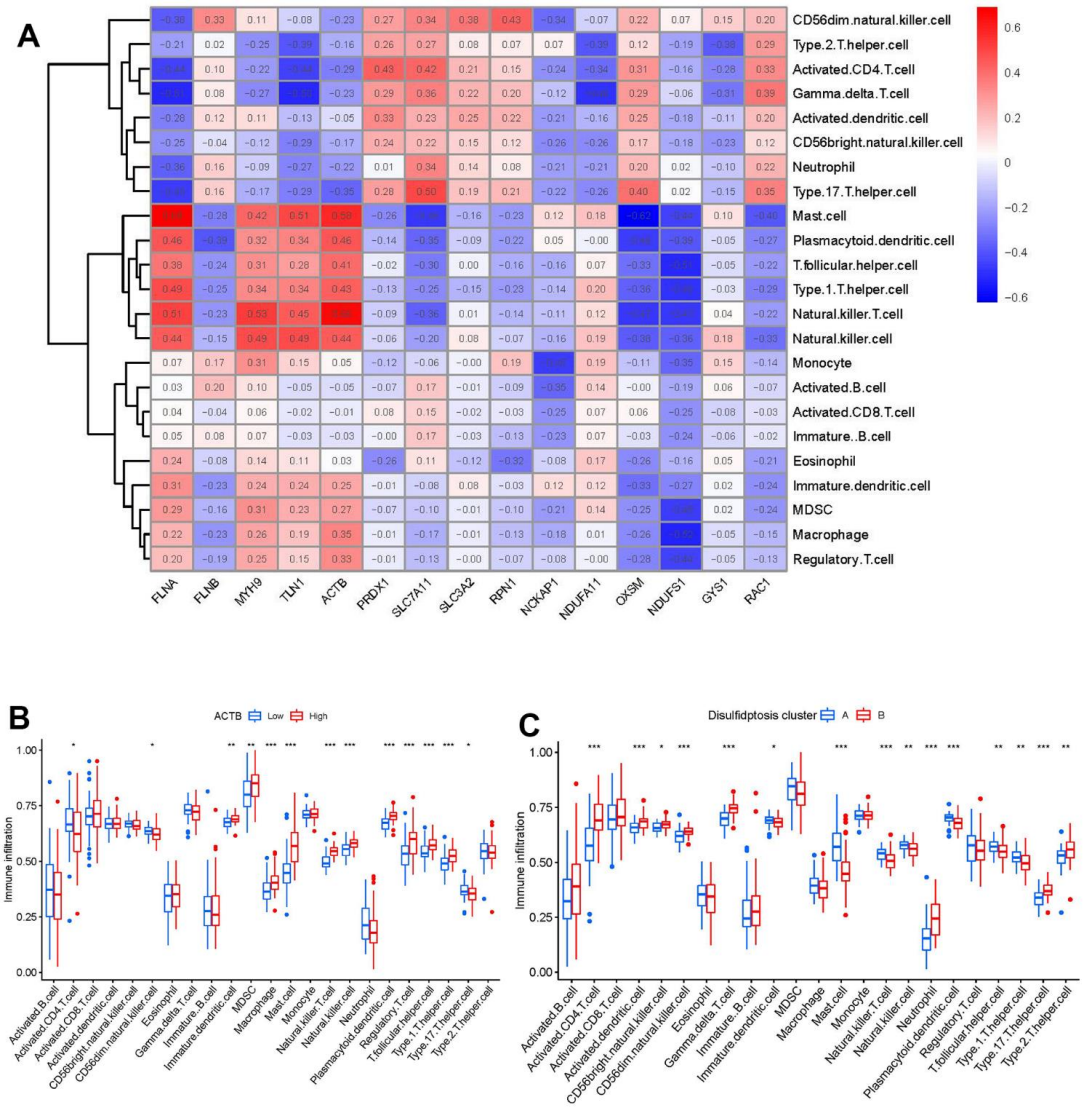
**Single sample gene set enrichment analysis.** (**A**) Correlation between immune cell infiltration and the 15 significant disulfidptosis modulators. (**B**) Difference in the abundance of infiltrating immune cells between high and low ACTB protein expression groups. (**C**) Differential immune cell infiltration between clusterA and clusterB. *p < 0.05, **p < 0.01, and ***p < 0.001.

Then, we investigated the link between immune cells and 15 significant disulfidptosis regulators using ssGSEA to determine the immune cell abundance in GC samples. We noticed a positive correlation between ACTB and many immune cells (Figure 7A). Between patients with high and low ACTB expressions, we examined the variations in immune cell infiltration. According to the findings, GC patients with high ACTB expression had a higher enhanced immune cell infiltration than patients with low ACTB expression (Figure 7B). We discovered that clusterB was linked to activated CD4 T cell, activated dendritic cell, CD56bright natural killer cell, CD56dim natural killer cell, gamma delta T cell, neutrophil, Type 17 T helper cell, Type 2 T helper cell immunity, while clusterA was linked to the immunity of immature dendritic cell, mast cell, natural killer T cell, natural killer cell, plasmacytoid dendritic cell, T follicular helper cell, Type 1 T helper cell (Figure 7C).

### Disulfidptosis gene signature establishment through two distinct disulfidptosis gene patterns

**Figure 8:**
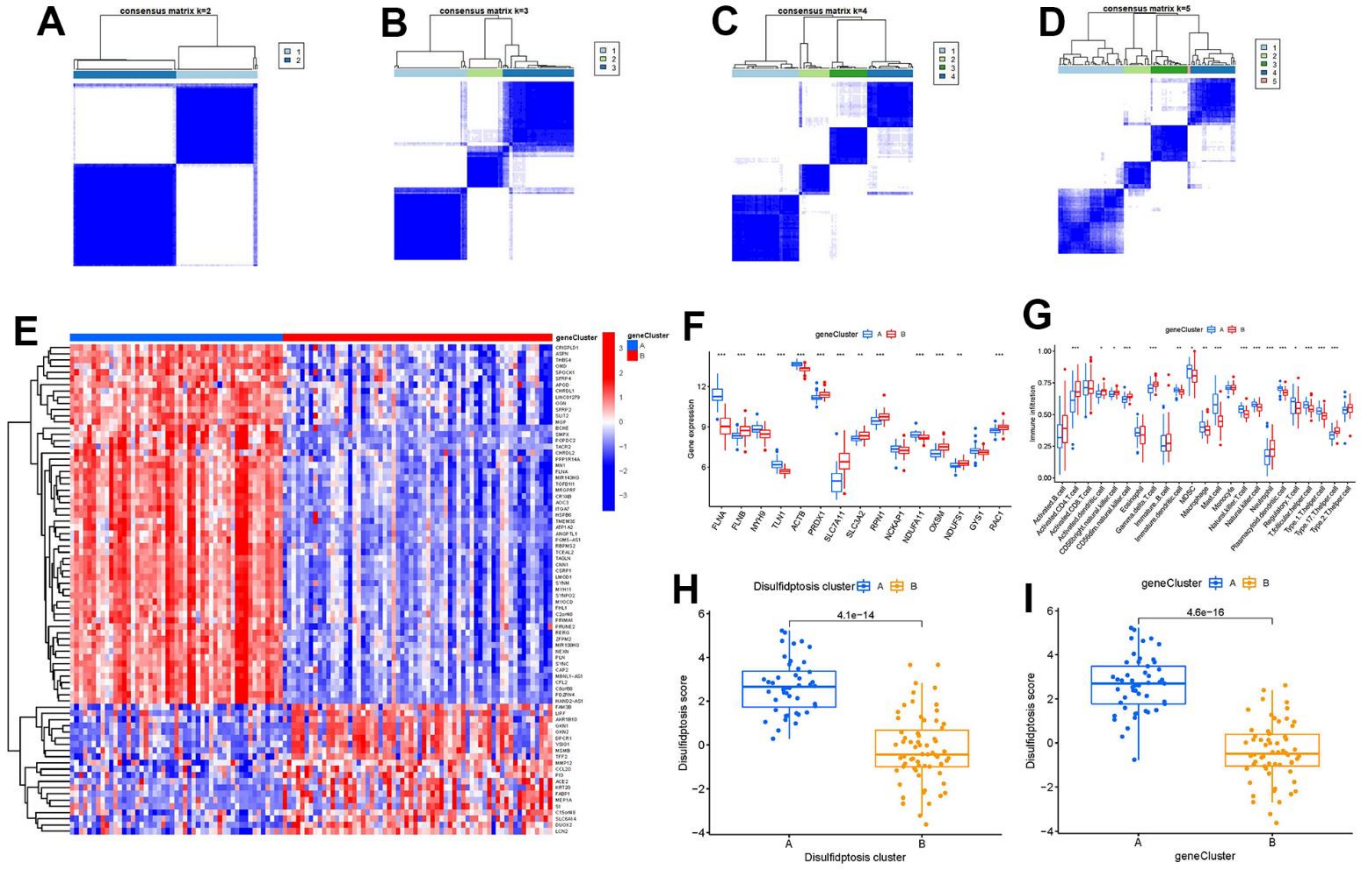
**Consensus clustering of the 79 disulfidptosis-associated DEGs in GC.** (**A**–**D**) Consensus matrices of the 79 disulfidptosis-associated DEGs for k = 2–5. (**E**) Expression heat map of the 79 disulfidptosis-associated DEGs in gene clusterA and gene clusterB. (**F**) Differential expression boxplots of the 15 significant disulfidptosis modulators in gene clusterA and gene clusterB. (**G**) Differential immune cell infiltration between gene clusterA and gene clusterB. (**H**) Differences in disulfidptosis score between clusterA and clusterB. (**I**) Differences in disulfidptosis score between gene clusterA and gene clusterB. *p < 0.05, **p < 0.01, and ***p < 0.001.

On the basis of the 79 disulfidptosis-associated DEGs, we classified the GC cases into several genomic subtypes using a consensus clustering technique in order to illuminate the disulfidptosis patterns. We discovered two unique disulfidptosis gene patterns (gene clusters A and B), which matched the sectionalization of disulfidptosis patterns (Figure 8A–8D). The expression levels of the 79 disulfidptosis-related DEGs in gene clusterA and gene clusterB were shown in Figure 8E. Immune cell infiltration levels and the expressions of 15 significant disulfidptosis modulators between gene clusterA and gene clusterB were also analogous to those in the disulfidptosis patterns (Figure 8F, 8G). The accuracy of our sectionalization by the consensus clustering method was once again confirmed by these results. PCA techniques were used to quantify the disulfidptosis patterns by calculating the disulfidptosis scores for each sample between the two different disulfidptosis patterns or disulfidptosis gene patterns. In comparison to clusterB or gene clusterB, we discovered that clusterA or gene clusterA displayed a higher disulfidptosis score, showing that clusterA or gene clusterA may be more correlated with GC (Figure 8H, 8I).

### Disulfidptosis cluster role for GC identification

**Figure 9:**
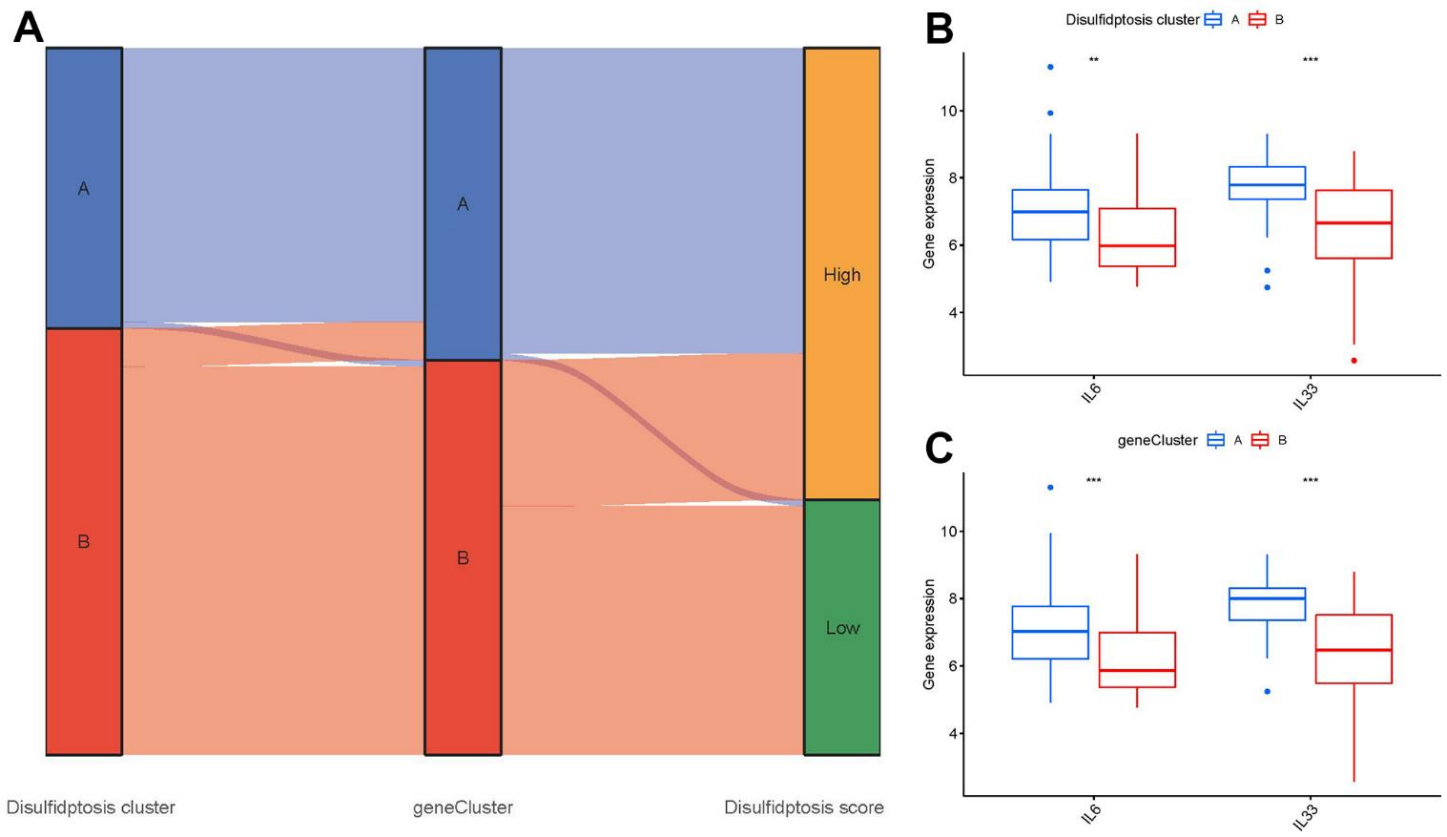
**Role of disulfidptosis patterns in distinguishing GC.** (**A**) Sankey diagram showing the relationship between disulfidptosis patterns, disulfidptosis gene patterns, and disulfidptosis scores. (**B**) Differential expression levels of immune-related genes between clusterA and clusterB. (**C**) Differential expression levels of immune-related genes between gene clusterA and gene clusterB. **p < 0.01, and ***p < 0.001.

The relationship between disulfidptosis scores, disulfidptosis patterns, and disulfidptosis gene patterns was shown in virtue of a Sankey diagram (Figure 9A). To investigate the relationship between disulfidptosis patterns and GC, we examined the correlation between disulfidptosis patterns and IL6, IL33 which have close association with natural killer cell immunity. We found that clusterA or gene clusterA had higher levels of IL6 and IL33 expression than clusterB or gene clusterB, indicating that disulfidptosis patterns was strongly connected with GC defined by natural killer cell (Figure 9B, 9C).

## DISCUSSION

Gastric cancer (GC) is a malignant gastrointestinal tumor that is characterized by strong chemotherapeutic resistance to current therapies due to the highly heterogeneous and immature phenotype of cancer cells during tumourgenesis and development, thus adversely affecting health and life quality [16]. Cases of GC are expected to increase to about 1.8 million by 2040, while the number of deaths due to GC worldwide will reach 1.3 million each year [17]. Due to the insidious nature of symptoms, patients used to be diagnosed with advanced and unresectable GC [18]. Therefore, good prognosis largely depends on early diagnosis and effective treatment of GC. Existing researches have confirmed that sulfide-related protein SLC7A11 could reversely regulate ferroptosis of GC cells and thus promote the progression of GC, suggesting disulfidptosis may exist in the pathological process of GC [8]. It is uncertain if disulfidptosis regulators have a crucial role in GC. The current work focused on exploring the function of disulfidptosis regulators in GC.

Firstly, totally 15 significant disulfidptosis regulators were identified from 17 disulfidptosis regulators via differential expression analysis between GC samples and controls, including FLNA, FLNB, MYH9, TLN1, ACTB, PRDX1, SLC7A11, SLC3A2, RPN1, NCKAP1, NDUFA11, OXSM, NDUFS1, GYS1, RAC1. According to a constructed RF model to predict the occurrence of GC, ACTB was chosen as diagnostic disulfidptosis regulators, which has a high AUC value of 0.994. Thereafter, we developed a nomogram model of the hub gene ACTB, which has been assessed using the DCA curve to generate benefits for GC patients by means of decisions based on the nomogram model.

Numerous studies have confirmed that sulfide-related proteins take an active part in regulating the carcinogenesis process and tumor metabolism balance in GC. For example, SLC7A11 (cystine transporter solute carrier family 7 member 11, also known as xCT), mediates cystine uptake and promotes disulfidptosis under glucose starvation [6]. As shown in Figure 2J, SLC7A11 exhibited lower expression level in GC samples. Existing study has confirmed that GC patients with unbalanced expression of SLC7A11-AS1/xCT axis had a poor prognosis and relatively poor response to chemotherapy [19]. SLC7A11 also acts as an epigenetic co-factor to interfere with the stemness of GC cells [20]. RAC1 (Ras-related C3 botulinum toxin substrate 1) is a small GTPase, the upregulation of which activates disulfidptosis [6]. RAC1 was reported to serve as an independent tumor diagnostic biomarker and survival predictor, and knockdown of RAC1 exerted suppressive effect on epithelial-mesenchymal transition in GC cells [21]. PRDX1 (Peroxiredoxin 1) serves as a known modulator of ROS in regulating oxidative stress [22], which could regulate epithelial-mesenchymal transition in GC cells [23]. Existing study has reported that OXSM is mitochondrial 3-Oxoacyl-ACP synthase targeting miR-338-3p [24], which has important functions in regulating epigenetic mediation of DNA methylation leading to GC pathogenesis [25]. Actin cytoskeleton vulnerability to disulfide stress mediates disulfidptosis; actin cytoskeleton proteins show strong susceptibility to disulfide stress induced by overaccumulation of intracellular disulfide molecules, and aberrant disulfide bonding among actin cytoskeleton proteins can trigger actin network collapse and disulfidptosis [6]. The actin cytoskeleton proteins ACTB (actin) and FLNA (filamin-A) containing multiple cysteine sites with glucose starvation-induced disulfide bonds, serve as disulfidptosis modulators exerting synergetic effects with glucose starvation to induce disulfidptosis [6]. Study has confirmed that actin cytoskeleton rearrangement and epithelial-mesenchymal transition process play an important role in cell migratory and invasive abilities of GC [26]. Our present study indicated that GC patients with high ACTB expression had a higher enhanced immune cell infiltration than patients with low ACTB expression (Figure 7B). It has been verified that FLNA takes part in the regulation of migration and invasion of GC cell by promoting degradation of MMP-9 *in vitro* [27]. To the best of our acknowledge, these disulfidptosis regulators may be crucial in the onset and progression of GC.

Growing research indicates that natural killer cells are crucial to the development of GC pathogenesis [28]. We noticed that cluster A was highly correlated with abundant immune cell infiltration of natural killer cell, which has close connection with GC (Figure 7C). Natural killer (NK) cells as robust prognostic biomarkers, play a crucial regulatory role in preserving immunological homeostasis [29] and opening up new avenues for natural killer cell-based GC immunotherapy is beneficial for preventing the development of GC [30]. IL6 and IL33 were closely associated with natural killer cell. IL6 plays a vital role in inhibiting activation of NK cells [31], which is involved in regulating GC cell proliferation and invasion [32]. IL-33 primarily activates natural killer cell [33], which plays an important role in regulating angiogenesis and cancer progression in GC [34]. In the current study, we excavated two different disulfidptosis clusters (clusterA and clusterB) based on the 15 significant disulfidptosis regulators as well as two distinct disulfidptosis gene patterns (gene clusterA and gene clusterB) on the basis of the 79 disulfidptosis-associated DEGs. ClusterA and gene clusterA were closely correlated with the natural killer cell immunity and displayed higher expression levels of IL6 and IL33. Additionally, PCA methods were used to determine the disulfidptosis scores for each sample between the two different disulfidptosis clusters or disulfidptosis gene clusters in order to quantify the disulfidptosis signatures. We discovered that compared to clusterB or gene clusterB, clusterA or the gene clusterA displayed a higher disulfidptosis score. Our study demonstrated that the patients in clusterB with low-risk disulfidptosis scores had a greater abundance of activated CD4 T cell. Previous studies have shown that abundant immune cells are infiltrated in the tumor microenvironment of GC, and the number and phenotype of immune cell subpopulations in GC tissues are closely correlated with the development and prognosis of GC patients [35]. CD4 T cells are a special type of T cells targeting tumor cells in various ways, which can directly eliminate tumor cells through cytolysis, or indirectly kill tumor cells by increasing the number of B Cells and CTL (Cytotoxic T Lymphocytes) responses [36, 37]. Existing study reveals that DNA damage repair and immunogenic tumor microenvironment in GC could be characterized by activated subsets of CD4 T cells [38].

Our findings support the participation of these disulfidptosis regulators in GC and offer novel insight into their function in the GC etiology, further supporting the idea that disulfidptosis modulators may be crucial in the progression of GC. That is to say, targeting these disulfidptosis targets may be a promising therapeutic approach for modulating the balance of tumor microenvironment in GC. To the best of our knowledge, this study is the first time to identify disulfidptosis-related diagnostic clusters and immune landscapes in GC. However, there exist some limitations to this study. Further experimental validations are necessary to confirm the specific mechanisms by which disulfidptosis-related genes affect specific pathways and the tumor microenvironment.

## CONCLUSIONS

Our current study generally tested 15 diagnostic disulfidptosis regulators and built a nomogram model that accurately predicted the occurrence of GC. Then, using the 15 disulfidptosis regulators, we verified two disulfidptosis signatures and discovered that clusterA or gene clusterA may be more linked with GC. To the best of our knowledge, this study is the first to pinpoint immunological landscapes and diagnostic clusters associated with disulfidptosis in GC.

## Supplementary Material

Supplementary Tables
