# Supplementary material for: Unravelling diagnostic clusters and immune landscapes of disulfidptosis patterns in gastric cancer through bioinformatic assay
Source: Aging (Albany NY). 2023 Dec 27;15(24):15434–50. doi: 10.18632/aging.205365 (PMC10781506; doi:10.18632/aging.205365)
Supplement: Supplementary Tables [file aging-15-205365-s001.pdf]

SUPPLEMENTARY TABLES

Supplementary Table 1. The detailed information of GO enrichment analysis in Figure 2R.

ONTOLOGY	ID	Description	p-value	p.adjust
BP	GO:0070527	platelet aggregation	7.77E-10	5.58E-07
BP	GO:0034109	homotypic cell-cell adhesion	3.94E-09	1.41E-06
BP	GO:0030168	platelet activation	9.73E-08	1.88E-05
BP	GO:0007596	blood coagulation	1.42E-07	1.88E-05
BP	GO:0007599	hemostasis	1.55E-07	1.88E-05
BP	GO:0050817	coagulation	1.57E-07	1.88E-05
BP	GO:0007016	cytoskeletal anchoring at plasma membrane	2.70E-05	0.002770637
BP	GO:0034329	cell junction assembly	3.31E-05	0.002966568
BP	GO:0034330	cell junction organization	6.80E-05	0.004887264
BP	GO:0007229	integrin-mediated signaling pathway	7.07E-05	0.004887264
CC	GO:0005925	focal adhesion	8.18E-09	3.07E-07
CC	GO:0005924	cell-substrate adherens junction	8.61E-09	3.07E-07
CC	GO:0030055	cell-substrate junction	9.21E-09	3.07E-07
CC	GO:0042470	melanosome	1.03E-06	2.06E-05
CC	GO:0048770	pigment granule	1.03E-06	2.06E-05
CC	GO:0001726	ruffle	7.08E-06	0.000118024
CC	GO:0005903	brush border	5.35E-05	0.000763911
CC	GO:0005938	cell cortex	6.96E-05	0.000835652
CC	GO:0005884	actin filament	7.52E-05	0.000835652
CC	GO:0030863	cortical cytoskeleton	8.57E-05	0.00085742
MF	GO:0045296	cadherin binding	1.08E-07	1.16E-05
MF	GO:0050839	cell adhesion molecule binding	1.21E-06	6.46E-05
MF	GO:0015175	neutral amino acid transmembrane transporter activity	0.00026672	0.007652178
MF	GO:0003779	actin binding	0.000286063	0.007652178
MF	GO:0051015	actin filament binding	0.000458475	0.00981136
MF	GO:0099516	ion antiporter activity	0.000936647	0.016703538
MF	GO:0048365	Rac GTPase binding	0.001322735	0.020218942
MF	GO:0015171	amino acid transmembrane transporter activity	0.001519457	0.020322732
MF	GO:0015297	antiporter activity	0.001998362	0.023758299
MF	GO:0005200	structural constituent of cytoskeleton	0.002861299	0.030615899

Supplementary Table 2. The detailed information of KEGG enrichment analysis in Figure 2S.

Term	p-value	p.adjust
Focal adhesion	2.70E-07	2.29E-05
Salmonella infection	7.79E-07	3.31E-05
Pathogenic Escherichia coli infection	1.14E-05	2.28E-04
Diabetic cardiomyopathy	1.29E-05	2.28E-04
Proteoglycans in cancer	1.34E-05	2.28E-04
Regulation of actin cytoskeleton	1.71E-05	2.42E-04
Amyotrophic lateral sclerosis	1.26E-04	0.001526033
Non-alcoholic fatty liver disease	1.94E-04	0.002061129
Tight junction	2.50E-04	0.002363637
Ferroptosis	4.23E-04	0.003598003

Supplementary Table 3. The detailed information of GO enrichment analysis in Figure 6H.

ONTOLOGY	ID	Description	p-value	p.adjust
BP	GO:0006936	muscle contraction	3.05E-10	4.44E-07
BP	GO:0003012	muscle system process	7.47E-09	5.45E-06
BP	GO:0006937	regulation of muscle contraction	2.63E-06	0.001278253
BP	GO:0090287	regulation of cellular response to growth factor stimulus	1.40E-05	0.005097376
BP	GO:0007517	muscle organ development	2.36E-05	0.00688819
BP	GO:0090257	regulation of muscle system process	3.55E-05	0.008035378
CC	GO:0043292	contractile fiber	7.08E-10	9.13E-08
CC	GO:0030016	myofibril	8.07E-09	5.21E-07
CC	GO:0030017	sarcomere	5.42E-08	2.33E-06
CC	GO:0030018	Z disc	3.74E-07	1.21E-05
CC	GO:0031674	I band	6.49E-07	1.68E-05
CC	GO:0030055	cell-substrate junction	0.000122462	0.002256807
MF	GO:0008307	structural constituent of muscle	1.44E-05	0.002218919
MF	GO:0003779	actin binding	2.37E-05	0.002218919
